# Preparation and Characterization of Hot Melt Copolyester (PBTI) Ultrafine Particles and Their Effect on the Anti-Pilling Performance of Polyester/Cotton Fabrics

**DOI:** 10.3390/polym10101163

**Published:** 2018-10-18

**Authors:** Zhichao Huang, Wenxing Chen

**Affiliations:** Key Laboratory of Advanced Textile Materials and Manufacturing Technology, Ministry of Education, Zhejiang Sci-Tech University, Hangzhou 310018, China

**Keywords:** hot-melt copolyester, ultrafine particles, anti-pilling

## Abstract

An ultrafine particle aqueous-phase system of hot melt copolyester was prepared by an inverse emulsion–precipitation method. Laser particle size analysis showed that the diameter of the obtained copolyester particles was mostly distributed between 20 and 100 nm. The structure of the copolymer was characterized by FT–IR and ^1^H-NMR, and the melting point of the particles was determined to be 125 °C, as measured by differential scanning calorimetry (DSC). Intrinsic viscosity analysis showed that the particle intrinsic viscosity decreased by 6.73% compared with that of the original copolyester. Polyester/cotton woven fabrics were padded with the ultrafine copolyester particles at different concentrations, and the corresponding SEM showed that the fibers were well bonded to each other. The pilling test results showed that these ultrafine copolyester granules improved the pilling performance of the polyester/cotton woven fabrics to a grade of 4.5–5.

## 1. Introduction

With the improvement of people’s living standards, environmental protection requirements are becoming increasingly more stringent. Nontoxic, nonpolluting, hot melt adhesives [1,2] with high bonding strengths have attracted more and more attention. Polybutylene terephthalate-co-polybutylene isophthalate *(*PBTI) is a kind of copolyester based on polybutylene terephthalate (PBT) and modified with polybutylene isophthalate (PBI). Because of its good crystallinity, good compatibility, and melting point control, it is widely used in hot melt fibers, masterbatch, and nonwoven fabrics. Compared with other types of hot melt adhesives [3], it has broader application prospects, owing to its excellent fabric feel, low price, and resistance to washing, sand washing, and steam pressing.

A polymeric ultrafine particle material [4] refers to a polymeric material or a polymer composite material whose particles have a diameter ranging from nanometers to micrometers and a spherical shape or other geometry. Polymer particles are mainly used in the surface treatment of coatings, paper, adhesives, plastic additives, and other materials. The method for preparing polymer microparticles by using a polymer as the raw material mainly includes an emulsion–solidification method, a complex coacervation method, and the precipitation of a homogeneous polymer solution. For example, Corre et al. [5] prepared polylactic acid microsphere materials by an emulsion–solidification method using two polylactic acid blends with different molecular weights (2000 and 9000) as the film; Bayomi et al. [6] prepared complex coacervate microcapsules of chitosan and casein by complex coacervation. Hou and Lloyd [7] prepared uniform sized nylon particles in a formaldehyde solution by precipitation of a homogeneous polymer solution. These methods produced ultrafine particles of polymers, but the preparation of ultrafine particles of PBTI copolyester has not been reported. The size of the powder obtained by the conventional swelling and milling method [8] does not reach the ultrafine level, so it is worth pursuing the preparation of ultrafine particles of PBTI.

The pilling phenomenon of fabrics is a common problem in textiles and garments, affects the aesthetics, and greatly reduces the added value of the products. At present, the traditional resin finishing method [9,10,11,12] is still the main one used. For example, Montazer [13] et al. applied resin and a cross-linking agent to reduce the pilling performance of fabrics; Cao [14] et al. used the anti-pilling agent ATP for fabric anti-pilling finishing; Xu [15] et al. used a synthetic silicone-modified polyurethane finishing agent for anti-pilling finishing; Tusief et al. [16] used a special anti-pilling finishing agent on a dyed polyester/cotton blend fabric for anti-pilling treatment. The main disadvantages of these methods include one or several such problems as the safety of the cross-linking agent, the poor durability, and the poor fabric feel. Recently, it has been reported that Rombaldoni [17] et al. used an Hexamethyldimethicone(HMDSO)–O_2_–Ar gas mixture to form a film on a wool knit fabric by vapor deposition to acheive anti-pilling. Kowalczyk et al. [18] used gold/silver nanopowders to reduce the pilling performance of polyester/cotton blend fabrics (67/33) by the sol–gel method. The results showed that the anti-pilling performance of a polyester/cotton blended fabric after treatment with SiO_2_/Al_2_O_3_ combined with gold/silver nanopowder was greatly improved. Although this method works well, the deposition efficiency is not high, which is not conducive to mass production. Noreen et al. [19] treated the polyester/cotton blend fabric (52/48) with bio-polishing cellulase. The results showed that the best effect was obtained at a temperature of 55 °C and a pH of 5.0. The disadvantage of this method is that it is not time-sensitive. Li [20] and others used different proportions of low-melting-point fiber and soybean protein fiber to improve the anti-pilling performance of fabrics. The results showed that the anti-pilling performance was improved after heat treatment, but the disadvantage was that the higher the content of the mixed low-melting fiber, the higher the cost, the worse the hand feel.

In this work, sulfuric acid was used as the solvent, n-hexane was the continuous phase, water was the dispersed phase, alkyl phenol ether sulfosuccinate sodium salt (OS) was the emulsifier, and polyethylene glycol (PEG-400) was the dispersant. The PBTI hot melt copolyester ultrafine particle aqueous-phase system was prepared by an inverse emulsion–precipitation method. A polyester/cotton fabric was finished by the dip immersion method to improve its anti-pilling performance. The method could improve the pilling performance of the fabric and could overcome the problem of excessive formaldehyde in the cross-linking reaction that occurs with the conventional resin finishing method. In the present method, the hot melt particles do not undergo a chemical reaction during the thermal bonding process, generally do not yield related byproducts, and the material itself is safe. Secondly, the macromolecular structure of the PBTI copolyester and ordinary polyester is similar to that of polyester, so the affinity is strong, and the bond strength is high, and the problem of poor durability by the conventional method is improved. Also, in the traditional method, when the coating is thick, the fabric feels poor. Differently from conventional method, the presented method can reduce the area of surface adhesion and then reduce bonding. According to the current literature, a method using copolyester hot melt adhesives for improving the pilling resistance of fabrics has not been described. Therefore, the application of PBTI hot melt copolyester to the finishing of fabrics is a worthwhile attempt.

## 2. Experimental

### 2.1. Materials

The PBTI hot melt copolyester was produced in our laboratory, with a melting point of 125 °C and an intrinsic viscosity of 0.683 dL/g, and was prepared by esterification–polycondensation using terephthalic acid (PTA), isophthalic acid (IPA), and 1,4-butanediol (BDO). Blue polyester/cotton (65/35) was provided by Hangzhou Yihang Textile Co., Ltd., Hangzhou, China. The specification was 265 g/m^2^. The emulsifier OS (chemical pure grade) and the dispersant PEG-400 (chemical pure grade) were purchased from Hai’an Petrochemical Plant, Nantong, China. Concentrated sulfuric acid (chemical pure grade) was purchased from Zhejiang Sanying Chemical Reagent Co., Ltd. (Jinhua, China), and n-Hexane (chemical pure grade) was purchased from Shanghai Lingfeng Chemical Reagent Co., Ltd., (Shanghai, China). Tetrachloroethane (chemical pure grade) was supplied by Shanghai Lingfeng Chemical Reagent Co., Ltd., China. Phenol (chemical pure grade) was purchased from Hangzhou Gaojing Fine Chemical Co., Ltd. (Hangzhou, China). Sodium hydroxide (chemical pure grade) was obtained from Zhejiang Dafang Chemical Reagent Factory (Hangzhou, China).

### 2.2. Processing

#### 2.2.1. Preparation of Copolyester Particles

Five grams of frozen and pulverized homemade low-melting hot melt copolyester was added to an Erlenmeyer flask containing 100 mL of 80% H_2_SO_4_ and magnetically stirred for 6 h until completely dissolved. Then, 75 mL of deionized water, 0.5 mL of OS surfactant, 0.2 mL of PEG-400 dispersant, and 200 mL of n-hexane were sequentially added to a three-necked flask, and a stable inverse emulsion system was prepared by high-speed stirring. After that, the dissolved copolyester solution was slowly added dropwise to the three-necked flask under high-speed stirring to obtain a uniform emulsion of the particles, and, after standing for 0.5 h, the lower phase of the suspension was taken, and an appropriate amount of sodium hydroxide was added thereto. Finally, the solution was diluted to 1000 mL in a volumetric flask with deionized water. A part of the liquid was centrifuged, washed with water, and lyophilized to obtain a copolyester powder.

#### 2.2.2. Anti-Pilling Finishing of Polyester/Cotton Woven Fabrics

A polyester/cotton woven fabric with a diameter of 12 cm was immersed in liquid suspensions with particle concentrations of 0.25 g/L and 0.5 g/L; the bath ratio was 1:50, and the liquid ratio was 120%. The padded sample was placed in a 60 °C oven for 3 h and then heated at 120 °C for 4 min. The prepared sample was finally washed three times and then dried at 60 °C for 3 h.

### 2.3. Characterization

Surface morphology was measured by a JEOL TM4000 SEM, Hitachi, Tokyo, Japan.

Particle size testing was carried out by a Marvin Zetasizer, Nano ZS laser particle size analyzer from Malvern, Worcestershire, UK.

Infrared spectroscopy (FT–IR) test: The sample was first hot-melt pressed into a film and then analyzed using a reflective accessory in a Nicolet 5700 Fourier infrared spectrometer, Thermo Fisher Scientific, Waltham, MA, USA.

Nuclear magnetic resonance (^1^H-NMR) test: The sample was dissolved in a solvent consisting of CDCl_3_/CF_3_COOD using an FTNMR Digital NMR spectrometer from Bruker, BioSpin Co., Rheinstetten, Germany, with tetramethylsilane (TMS) as an internal standard.

Differential Scanning Calorimetry (DSC): The sample was vacuum-dried at 105 °C for 24 h and then tested with Q20 DSC from Waters, Inc., Milford, MA, USA. The nitrogen flow rate was 20 mL/min. First, the temperature was rapidly increased from room temperature to 180 °C, which was maintained for 5 min to eliminate the heat history; then, liquid nitrogen quenching was performed. The temperature was then raised to 150 °C at a heating rate of 20 K/min.

Intrinsic viscosity test: The intrinsic viscosity [η] was measured by an Ubbelohde viscometer from China Hangzhou Zhuoxiang Technology Co., Ltd., Hangzhou, China. The solvent was a 1:1 mixture of phenol and tetrachloroethane, and the polymer was formulated into a solution at a concentration of 0.5 g/dL at 25 ± 0.1 °C. The molecular weight was calculated according to the Mark–Houwink equation. Chuah et al. determined the parameters of the equation and proposed the following expression:

(1) $$[\eta ]=8.2\times 10^{-4}{\bar{M}}_{w}^{0.63}$$

Pilling performance test: The woven fabric was tested by a Laizhou Textile Instrument Co., Ltd. (Laizhou, China) Model YG502 circular trajectory pilling machine, according to the standards of the People’s Republic of China (GB/T 4802.1-2008); the knitted fabric was analyzed by a Wenzhou Fangyuan Instrument Co., Ltd. (Wenzhou, China), YG511S-II rolling box pilling instrument, according to the standards of the People’s Republic of China (GB/T 4802.3-2008).

The mechanical performance was tested by a SHIMADZU AG-I universal material testing machine, Japan, using a 5 kN sensor. The fabric side of the yarn was pulled. The warp direction length was 10 cm, and the width was 2 cm. The elastic recovery rate was tested by a AG-I universal material testing machine according to the standards of the People’s Republic of China (FZ/T 01034-2008).

The gas permeability was tested by a Wenzhou Fangyuan Textile Instrument Co., Ltd. (Wenzhou, China), China, YG461E gas permeability tester, according to the standards of the People’s Republic of China (GB/T 5453.1-1997).

The stiffness was tested by a Laizhou Electronic Instrument Co., Ltd. (Laizhou, China), LLY-01B type electronic stiffness meter, according to the standards of the People’s Republic of China (GB/T 18318-2001).

## 3. Results and Discussion

### 3.1. Morphology and Distribution of Copolyester Particles

**Figure 1:**
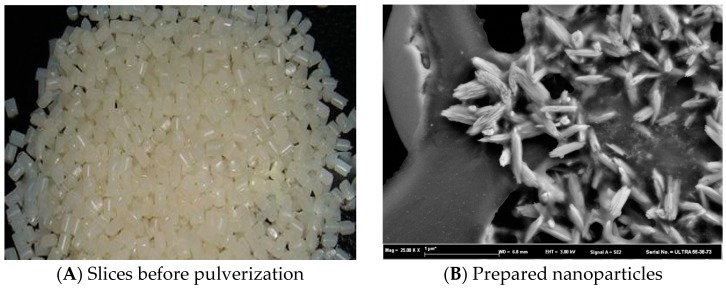
Photo of copolyester slices (**A**) and SEM of copolyester particles (**B**).

Figure 1A is a photo of copolyester slices and Figure 1B is a scanning electron micrograph of copolyester particles, showing that the prepared particles were uniform in size and exhibited an irregular rod-like or granular structure. It is speculated that the shape of the particles is related to the stirring speed of the copolymer (up to 3000 rpm) during the precipitation process. The fine aqueous-phase emulsion droplet environment is also an important factor in the formation of ultrafine particles.

**Figure 2:**
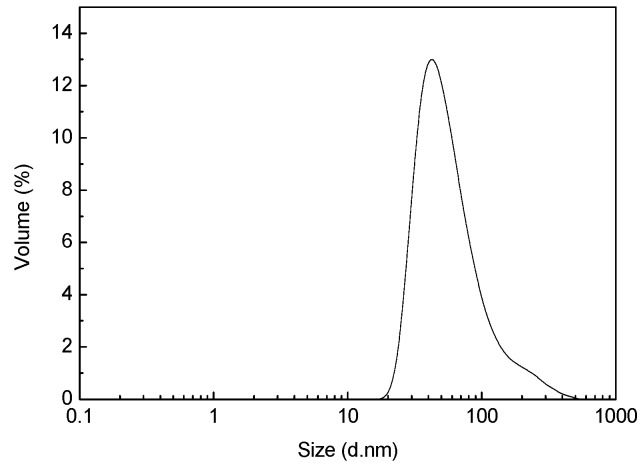
Particle size and distribution of the copolyester particles.

Figure 2 reveals the copolyester particle sizes and their distribution in the copolyester. Most of the polyester particles were found to have a particle size between 20 and 100 nm, and a small portion had a size of 100–800 nm. This shows that the particles obtained by this method can reach the nanometer scale with a uniform particle size distribution and can disperse well in an aqueous phase. This is in good agreement with Figure 1.

### 3.2. FT–IR

**Figure 3:**
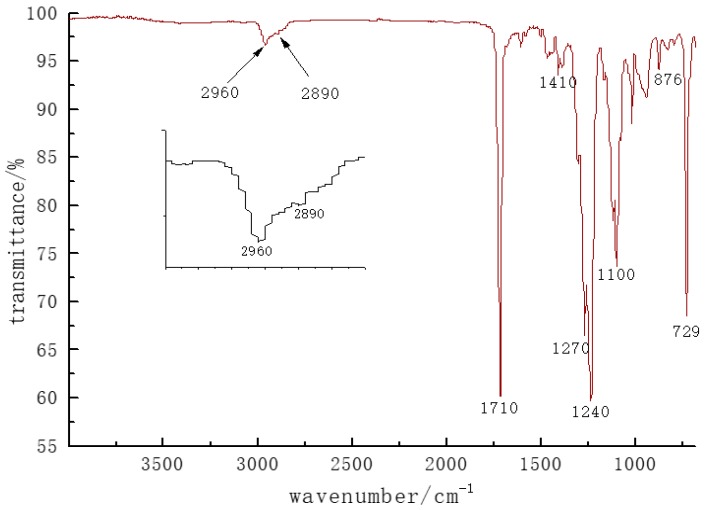
FT–IR of copolyester particles.

Figure 3 is an infrared spectrum of the PBTI hot melt copolyester. The sample showed a strong carbonyl stretching vibration peak at 1710 cm^−1^, a stretching vibration peak of CH_2_ at 2960 cm^−1^, a skeleton vibration peak of the benzene ring at 1410 cm^−1^, and stretching vibration peak of typical ester CO bond at 1240 cm^−1^ and 1270 cm^−1^. A symmetric stretching vibration peak—a peak of 1270 cm^−1^—indicates a characteristic peak of CO from a benzoic ester group, and an absorption peak of 1240 cm^−1^ is a characteristic peak of the CO connecting a benzoester group. At 1100 cm^−1^, we detected a C–O stretching vibration absorption peak of the ester group. The two peaks at 876 cm^−1^ and 1020 cm^−1^ correspond to the vibration absorption and the out-of-plane deformation vibration absorption of the phenyl–H in the phenyl group, respectively. At 729 cm^−1^, the rocking vibration of butanediol methylene was evident. The presence of methylene, p-phenyl, m-phenyl, and ester groups could be clearly detected from the infrared spectrum.

### 3.3. ^1^H-NMR

**Figure 4:**
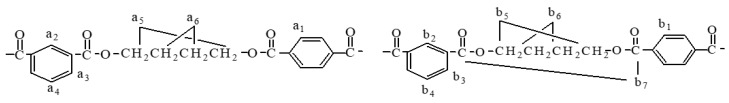
C and H in different locations in the co-polyester chain.

Figure 4 shows hydrogen and carbon atoms at different positions on the copolyester chain. The labels a1, a2, a3, a3, a4, a5, and a6 are protons at different structural positions; b1, b2, b3, b3, b4, b5, b6, and b7 are carbon atoms at different structural positions.

**Figure 5:**
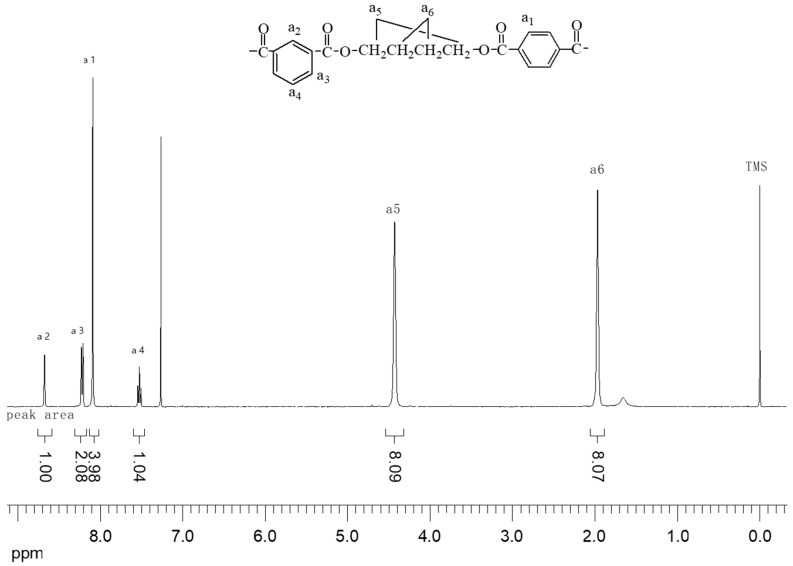
^1^H-NMR spectra of the sample polybutylene terephthalate-co-polybutylene isophthalate (PBTI).

Figure 5 is a ^1^H-NMR component analysis of PBTI. The peak at 0.000 is the internal standard TMS. The peaks at 1.655 and 1.658 are from the water molecule, H_2_O, which may be a residual water molecule after cleaning the sample tube. The peak at 1.968 is a hydrogen atom in the butadiene diester of the PBTI sample and is further away from the benzene ring, i.e., a6. A hydrogen atom in the butadiene diester is located at 4.430, which is closer to the benzene ring, that is, a5. The peak at 7.264 is the hydrogen atom on undeuterated chloroform. The peak at 7.5–8.7 is mainly from the hydrogen atom on the benzene ring (see Figure 3 and Figure 4), and that at 8.090 is from the hydrogen atom in the sample at the a1 position on the phenyl group. The remaining peak should be a hydrogen atom on the m-phenyl group. The peak at 7.506–7.545 is split into three peaks, indicating that the number of hydrogens at the adjacent carbon atom is two. This corresponds to the hydrogen atom at the a4 phenyl position, which meets this requirement. Similarly, the 8.29–8.229 region is split into two peaks, which correspond to the hydrogen atoms at the a3 position on the phenyl group. The remaining 8.675 unique peak is the hydrogen atom at the a2 position on the phenyl group [21,22].

According to the data in Table 1, the group ratio was inferred from the peak area corresponding to each hydrogen atom. Therefore, it could be inferred that the molar ratio of PTA, IPA, and 1,4-BDO was 1:1:2. It could be confirmed that the ratio of the PBT unit to the isobutylene PBI unit was 1:1.

### 3.4. Thermal Performance

**Figure 6:**
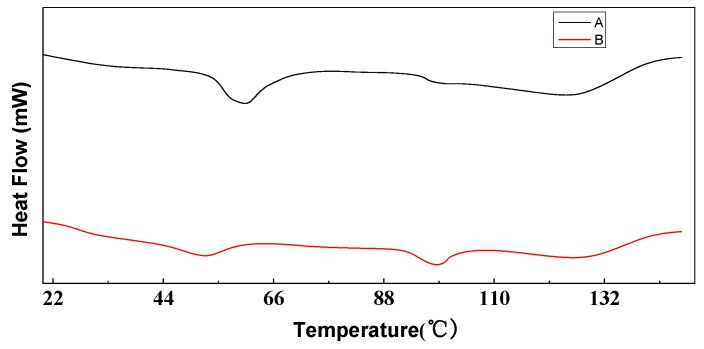
DSC curves (A: copolyester raw material, B: copolyester particles).

As can be seen from Figure 6, the melting point of the hot melt copolyester raw material was primarily constant after being turned into particles, and was about 125 °C. However, the melting peak of the particles became a typical double peak, which was mainly caused by the formation of melt recrystallization due to imperfect crystallization. The glass transition temperature Tg of the hot melt copolyester raw material was 60 °C, but the glass transition temperature of the particles was lowered to 55 °C, and passivation occured, mainly due to the decrease of the intrinsic viscosity and the increase of the specific surface area, which made the movement of the polymer segment easier. Therefore, the preparation process of the particles had a little effect on its melting point.

### 3.5. Intrinsic Viscosity of the Hot Melt Copolyester

Table 2 shows the change of intrinsic viscosity of the hot melt copolyester. From Table 2, the intrinsic viscosity of the hot melt copolyester is 0.683 dL/g, and that of the polyester nanoparticles is 0.637 dL/g. The intrinsic viscosity thus decreased by 6.73%. This was mainly due to the hydrolysis reaction of the hot melt copolyester macromolecules in the acidic environment during the dissolution process. The breakage of the ester group in the molecular chain caused the breakage of the macromolecular segment, and the entanglement between the molecular chains was reduced. The relative movement between segments became easier, so the intrinsic viscosity was reduced.

### 3.6. Anti-Pilling Performance of a Polyester/Cotton Woven Fabric

**Figure 7:**
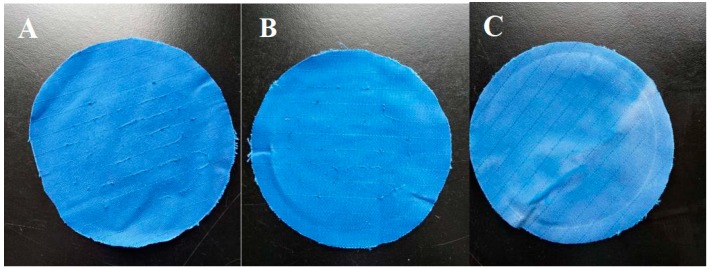
Pilling test on a polyester/cotton woven fabric (**A**): 0 g/L, (**B**) 0.25 g/L, (**C**) 0.5 g/L.

As shown in Figure 7, the anti-pilling performance of a polyester-cotton fabric treated with the low-melting-point hot-melt copolyester particles was remarkably improved. When the concentration of the immersion liquid was 0.5 g/L, no obvious pilling phenomenon was observed on the surface of the sample.

Table 3 shows the anti-pilling grades of the polyester/cotton fabric after 3, 20, and 50 washes, respectively. When the particle concentration reached 0.5 g/L, the fabric’s anti-pilling grade reached 4.5–5, which was 2.5–3 higher than that of the untreated fabric. Moreover, it did not change substantially after 50 washes, indicating that the durability of the treatment method was good, which is consistent with the results shown in Figure 7.

Description: The test measures the anti-pilling performance of fabrics by the circular path method according to the standards of the People’s Republic of China (GB/T 4802.1-2008). The anti-pilling level is divided into five levels: the fifth level is the best, and the first level is the worst.

**Figure 8:**
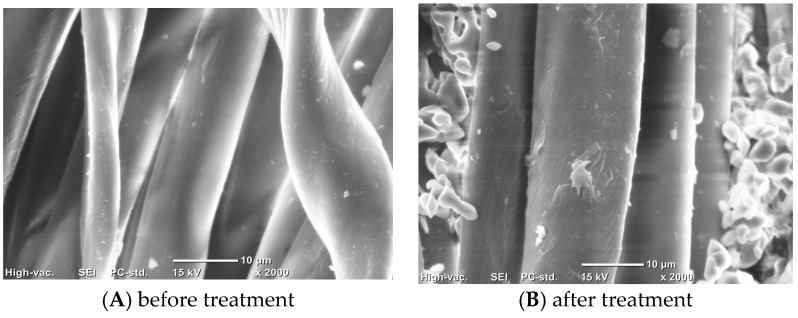
SEM of a polyester/cotton fabric before and after treatment.

Figure 8 is an SEM image of the surface of a polyester/cotton woven fabric before and after treatment. It shows that the hot melt particles were mainly deposited in the gaps between the fibers, and the fibers were bonded to each other after the hot melt processing, thereby objectively increasing the cohesion between the fibers or the yarns. Therefore, the fibers were not easily extracted from the yarn during the rubbing process. Thus, the pilling resistance of the fabric treated with the immersion liquid was remarkably improved. However, this was achieved in a different way with respect to the conventional resin finishing, which forms a film on the surface of the fabric to reduce friction.

### 3.7. Mechanical Properties of Polyester/Cotton Woven Fabrics

**Figure 9:**
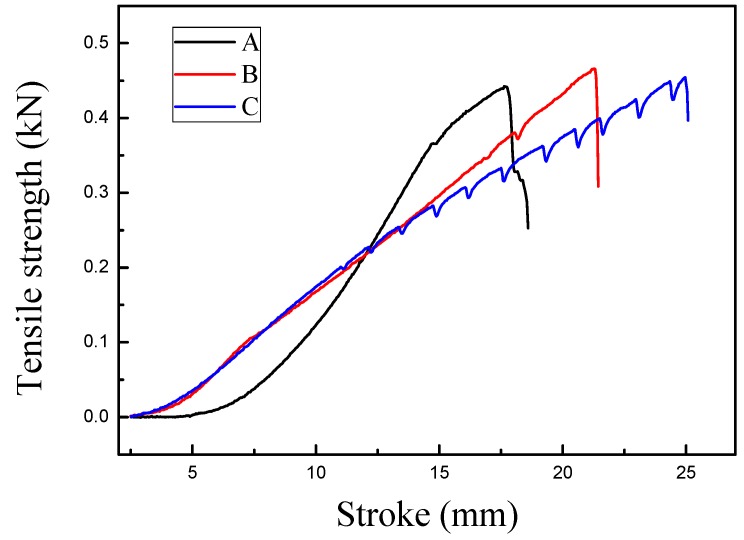
Tensile curves of polyester/cotton fabrics (A: 0 g/L, B: 0.25 g/L, C: 0.5 g/L).

Figure 9 A, B, and C represent the tensile curves of fabrics treated with different concentrations of particles. The curves show that the fabric breaking strength after the immersion treatment was slightly improved, and the elongation at break also increased. This is related to the formation of cross-links at the intersections or contacts of the low-melting-point hot-melt polyester particles with the fibers or yarns. It was also found that the C curve exhibited a wave shape. When the treatment immersion liquid reached a certain concentration, the tensile fracture of the fabric firstly destroyed the fiber or the slight cross-linking between the yarns, and then the physical structure was pulled or slipped. Finally, it caused the fabric to break. Therefore, the wave shape just reflects the result of the hot melt cross-linking of the particles.

**Figure 10:**
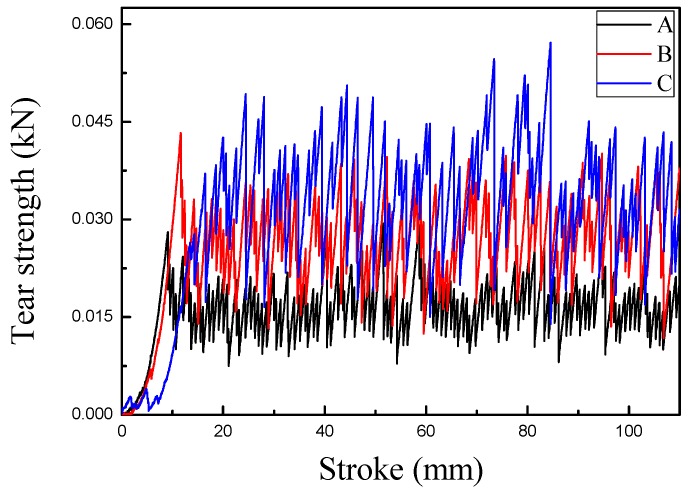
Tear curves of polyester/cotton fabrics (A: 0 g/L, B: 0.25 g/L, C: 0.5 g/L).

Figure 10, A, B, and C shows the tear curves of fabrics treated by different concentrations of particles. The curves show that the fabric tear strength after the immersion treatment greatly improved. The tear strength of sample C was larger than that of samples A and B, and its fluctuation was the largest. This can be explained similarly to the observations for sample C reported in Figure 9. The results were highly consistent.

### 3.8. Other Properties of the Treated Fabrics

Table 4 shows the gas permeability, flexibility, and resilience of polyester/cotton fabrics A, B, and C. The resilience of the fabrics treated with PBTI copolyester hot melt particles significantly improved as a result of the hot melt cross-linking of the particles. However, the air permeability was reduced, although the reduction was not large. The bending stiffness and flexural modulus of the fabric increased slightly, indicating that, as the particle concentration increased, the feel of the fabric slowly hardened. Therefore, in order to maintain the original hand feel of the fabric and simultaneously obtain better anti-pilling performance, it is important to control the particle concentration.

### 3.9. Anti-Pilling Mechanism

**Figure 11:**
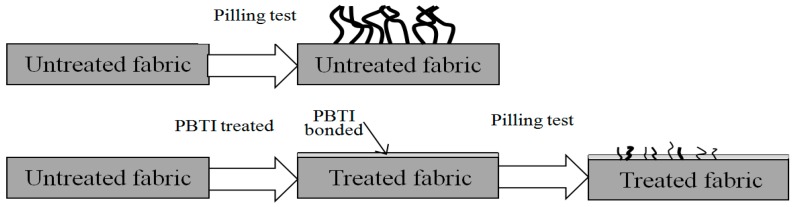
Diagram depicting the probable anti-pilling mechanism.

As discussed before, the anti-pilling performance of polyester/cotton woven fabrics was improved by padding ultrafine copolyester particles followed by heating. For the untreated fabric, after the pilling test, the inner fiber bundle was drawn out, and some fibers twined together, leading to an unpleasant pilling appearance. However, after PBTI was applied and heated, the fabrics could be bonded by PBTI, and, after the pilling test, fewer and thinner fibers were drawn out because of the blocking effect of PBTI. Figure 11 demonstrates the mechanism of this anti-pilling strategy by hot adhesion.

## 4. Conclusions

An ultrafine particle aqueous-phase system of hot melt copolyester was successfully prepared by an inverse emulsion–precipitation method. Most of the polyester particles with an irregular rod shape were found to have a particle size between 20 and 100 nm. FT–IR and ^1^H-NMR analyses showed that the PBT/PBI composition ratio in the hot melt copolyester PBTI was 1:1. Intrinsic viscosity analysis showed that the viscosity of the copolyester particles was 0.683 dL/g, which was 6.73% lower than that of the original copolymer, and DSC showed that the melting point of the particles was about 125 °C.

The anti-pilling performance of polyester/cotton woven fabrics was greatly improved to a 4.5–5 grade by using 0.5 g/L copolyester particles subjected to dipping and hot melting. SEM showed good adhesion between the fibers. The elastic recovery rate of the fabric was obviously increased, but the bending rigidity was also increased, and the gas permeability was slightly decreased. It is anticipated that the prepared polyester particles could be applied to other fabrics for improving their anti-pilling performance.

## Figures and Tables

**Table 1:** Numbers and peak areas of H in different locations of the co-polyester chain.

Chemical Shift (ppm)	H position on the Chain	H Number	Peak Area	Corresponding Peak Area of Each H
8.675	a_2_	1	1	1
8.209~8.229	a_3_	2	2.08	1.040
8.090	a_1_	4	3.98	0.995
7.506~7.545	a_4_	1	1.04	1.040
4.430	a_5_	4	8.09	2.023
1.968	a_6_	4	8.07	2.018

**Table 2:** Characteristic viscosity of the hot melt copolyester.

Solution	Glass Transition Temperature (°C)	Melting Point (°C)	Inherent Viscosity η/(dL/g)
Hot melt copolyester	60	125.4	0.683
Hot melt copolyester particles	55	125.1	0.637

**Table 3:** Anti-pilling grades of the polyester/cotton fabrics.

Polyester/Cotton Woven Fabric	Grade after Washing 3 Times	Grade after Washing 20 Times	Grade after Washing 50 Times
A (0 g/L)	2	2	2
B (0.25 g/L)	3.5	3.5	3–3.5
C (0.50 g/L)	4.5–5	4.5–5	4.5–5

**Table 4:** Other properties of the fabrics.

Polyester/Cotton Woven Fabric	Air Permeability (mm/s)	Bending Stiffness (cN·cm)	Flexuralmodulus (cN/cm^2^)	Resilience (%)
A (0 g/L)	53.09	0.57	2.48	39.28
B (0.25 g/L)	52.68	0.59	2.57	47.2
C (0.50 g/L)	51.33	0.60	2.61	49.5
